# Adenosine Triphosphate (ATP) and Protein Aggregation in Age-Related Vision-Threatening Ocular Diseases

**DOI:** 10.3390/metabo13101100

**Published:** 2023-10-20

**Authors:** Jack V. Greiner, Thomas Glonek

**Affiliations:** 1 Schepens Eye Research Institute of Massachusetts Eye & Ear Infirmary, Boston, MA 02114, USA; 2 Department of Ophthalmology, Harvard Medical School, Boston, MA 02115, USA; 3 Clinical Eye Research of Boston, Boston, MA 01890, USA; tglonek@rcn.com

**Keywords:** age, eye, lens protein, retinal protein, hydrotrope, hydration, water of hydration, organized water, ice-like

## Abstract

Protein aggregation is the etiopathogenesis of the three most profound vision-threatening eye diseases: age-related cataract, presbyopia, and age-related macular degeneration. This perspective organizes known information on ATP and protein aggregation with a fundamental unrecognized function of ATP. With recognition that maintenance of protein solubility is related to the high intracellular concentration of ATP in cells, tissues, and organs, we hypothesize that (1) ATP serves a critical molecular function for organismal homeostasis of proteins and (2) the hydrotropic feature of ATP prevents pathological protein aggregation while assisting in the maintenance of protein solubility and cellular, tissue, and organismal function. As such, the metabolite ATP plays an extraordinarily important role in the prevention of protein aggregation in the leading causes of vision loss or blindness worldwide.

## 1. Introduction

The most wide-spread age-related vision-threatening ocular diseases include age-related cataract, presbyopia, and age-related macular degeneration. A common finding among these three diseases, which ultimately may be experienced by all human beings with advancing age, is protein aggregation. Although protein aggregation can be a healthy advantage in the production of biogenic protein machines and organelles designed for intracellular and organismal gain of function, with aging, protein aggregation may result in loss of function. With aging, the risk of protein-aggregation-causing diseases increases. This is most likely because adenosine triphosphate (ATP) concentrations decline in cells during aging [1]. Age-related lens cataract is caused by light scattering resulting from aggregation of the long-lived lens crystallin proteins [2,3,4,5]. Presbyopia is caused by lens fiber cell stiffening secondary to protein aggregation [4,6,7]. Age-related macular degenerative changes are caused by the development of protein aggregates in and around the pigment epithelium and Bruch’s membrane [8,9,10,11].

Disease development as a result of protein aggregates is not limited to the aforementioned ocular diseases and can be considered a general process of the pathophysiology of disease [12,13]. The consequences of loss of solubility and protein aggregation include dysfunction or cessation of function(s) of enzymatic and structural proteins and can thus be detrimental to cellular, tissue, and organismal homeostasis. As such, an understanding of the mechanisms that prevent protein aggregation in normal healthy cells and tissues should provide insights into potentially promising clinical interventions to proactively prevent and/or mediate the consequences of protein aggregation. The ATP molecule meets the requirements consistent with the properties of a hydrotrope [14]. The recent reports on the hydrotropic function of this multifactorial metabolite ATP and its implications in terms of preventing protein aggregation and maintaining protein solubility are of particular interest [15,16,17,18,19,20].

This is a treatise on the three most profound age-related vision-threatening ocular diseases evaluating the interplay between the role of ATP and protein aggregation. The present perspective incorporates context and rationale from our previous works [19,21,22]. This perspective explores the relationship between the enigmatic millimolar concentration of ATP, which exceeds the micromolar concentration required for metabolic processes, and the maintenance of protein solubility and by extension prevention of protein aggregation.

## 2. Materials and Methods

Since ATP in high concentrations is reportedly functioning as a hydrotrope with dominant functions of preventing intracellular protein aggregation or maintaining protein solubility, the importance of these ATP functions is profound. With a paucity of studies linking ATP with protein aggregation coupled with the great importance of this finding in science and medicine [15,19,21], especially with its importance in vision-threatening ocular diseases, literature searches were conducted in PubMed. These literature searches used both controlled vocabulary and keywords, for the terms adenosine triphosphate, protein aggregation, cataract, crystalline lens, presbyopia, retina, optic nerve, and optic nerve head. No language or date restrictions were placed on the searches. A total of 685 articles were retrieved. The final search was run on 6 June 2023. After removing duplicates, title, abstract, and full-text screening were reviewed on the remaining 630 articles.

## 3. Results

The first reports of millimolar concentrations of ATP in an intact organized living tissue were in skeletal muscle in the 1970s using phosphorus-31 nuclear magnetic resonance (^31^P NMR) [23,24]. This high concentration was not unexpected since in this instance a high ATP concentration was deemed consistent with the known high metabolic demand of muscle tissue. Moreover, skeletal muscle cells and tissue possessed a large concentration of mitochondria, an organelle with a function of producing ATP efficiently and in high quantities [25]. Using the same ^31^P NMR technology, our discovery in 1981 of a similar and unexpectedly high concentration of ATP in the intact living crystalline lens [26] presented a conundrum, since the highly proteinaceous lens tissue functionally serves as a metabolically quiescent organ and was known to have a paucity of mitochondria [27]. Moreover, the lens also undergoes a process of de-nucleation with concomitant loss of mitochondria during maturation [27], though lenticular ATP concentration remains in the millimolar range throughout life [1].

### 3.1. ATP as A Hydrotrope

In 2017, studies on extracts of cells and tissue homogenates supported the postulation that ATP functions as a hydrotrope [15]. These findings, coupled with our ^31^P NMR studies on the intact living lens organ [26] and the interaction of intracellular ATP phosphate groups with water [28], generated a hypothesis that ATP in millimolar concentrations prevents protein aggregation due to its hydrotropic nature. Given that protein aggregation results in age-related ocular diseases (such as age-related cataract [19], presbyopia [6,22], and age-related macular disease [11]) and both lens and retina have been shown to have millimolar concentrations of ATP (2.3 mM [19] and 4.1 mM [29], respectively), it is conceivable that prevention of protein aggregation likely results from the high concentration of intracellular ATP. This concept eventually may be translational into disease prevention in those pathologies where protein aggregation has been identified as causal. If the basic assumptions of these studies with ATP functioning as a hydrotrope are correct [15,19], then there should be some relevant clinical correlation beyond the inevitable ophthalmic diseases of age-related cataract, presbyopia, and age-related macular degenerative diseases.

### 3.2. The Millimolar Concentration of ATP

The hydrotropic property of ATP, as it relates to protein aggregation, has been shown to be concentration-dependent [15], with the mean concentration in cells being ~4.4 mM [21]. Our proposed hypothesis, whereby ATP functions as a hydrotrope preventing protein aggregation [19], describes two potentially different pathophysiologies. First, the concentration of intracellular ATP levels declines below the millimolar levels found in healthy control cells and tissues [21]. Breaching this millimolar threshold may lead to protein aggregation. Secondly, the proteins in question may be altered in such a way that does not allow them to take advantage of an intracellular ATP environment that is comparable to the healthy controls. The resultant protein aggregation would be independent of an otherwise favorable intracellular ATP concentration. Mysteriously [30], all cells maintain very high ATP concentrations, much higher than those required for its previously known functions [15,18,31].

### 3.3. ATP and Protein Aggregation

As a standard biochemistry textbook delineates [31], the metabolite ATP is a multifaceted molecule having many important roles (Table 1). In addition, other novel roles for ATP have been proposed [32]. However, it appears that a major role of ATP in maintaining proteins in a disaggregated state has been overlooked [15,19]. This is especially evident in the crystalline lens where millimolar concentrations of ATP are found in both the cortex (4.1 mM) and the nucleus (1.3 mM) [33]. This is important considering the lens has another unique feature, that of having a concentration of protein comprised of crystallins that remain transparent for lens refraction and flexible for lens accommodation. The mature intralenticular fiber cells contain exceptionally high concentrations of crystallin proteins. Therefore, without ATP acting as a hydrotrope, the functionality of the lens organ with such a densely packed concentration of proteins would be expected to exhibit a greater propensity for protein aggregation resulting in the loss of transparency of the crystallin proteins in cataractogenesis and loss of flexibility of the lens in presbyopiogenesis [22].

### 3.4. The ATP Hydrotropic Layer

From our studies, we formulated the following concept: in the lens organ, the proteins are very close together; thus, the presence of an ATP hydrotropic layer on adjacent proteins will act to stabilize the interstitial water layer as the proteins approach each other. This stabilization of adjacent proteins forms a liquid-crystalline interstitial gel-like layer, where the participating water molecules are more firmly bound into stable locations between the adjacent protein layers. This stability is due to the polarizing effects of the negatively charged ATP phosphate groups comprising the triphosphate moiety. This gel-like layer then can act as a lubricating cushion between the adjacent protein molecules, keeping them separate.

### 3.5. The Hydrotropic Nature of ATP

**Figure 1:**
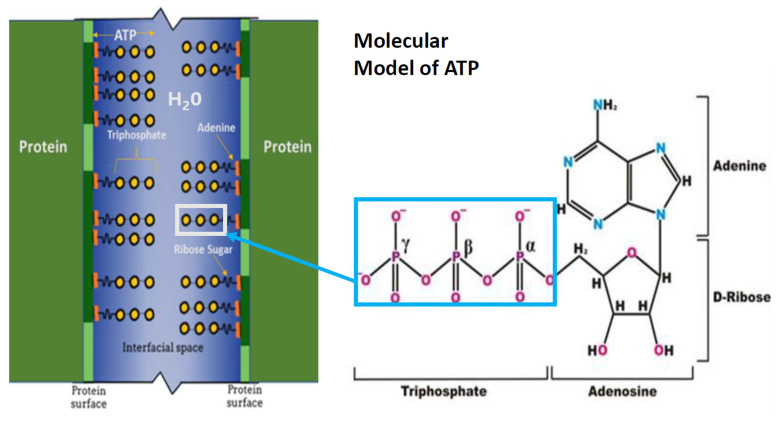
Schematic representation of interfacial space between adjacent protein molecules and ATP molecules. Interstitial water (blue) between the surfaces of two adjacent proteins (vertical green lines). The hydrophobic regions of adjacent proteins (dark green segments of vertical green lines) interact with ATP adenine residues (orange). ATP ribose sugar residue (short wavy black line) connects the hydrophobic adenine moiety with the hydrophilic ATP α, β, and γ-triphosphate residues (yellow circles). The triphosphate chain residue extends into the interfacial space, with the α-triphosphate residue in closest proximity to the ribose sugar residue. The molecular model represents ATP α, β, and γ triphosphate moiety joined to the ribose sugar attached to the adenine moiety. The box encloses the hydrophilic triphosphate moiety extending into interprotein water.

We hypothesize that the hydrotropic nature of ATP allows it to interact with intracellular water at the surface of a protein. This ATP–water–protein interaction organizes water molecules in the gel-like layer such that they exist in an ice-like structure. Such a coordination of water maintains a separation between adjacent proteins (Figure 1) [19]. This separation of proteins occurs presumably via ATP’s triphosphate group oxygen atoms complexing through hydrogen bonds with the interstitial inter-protein water hydrogen atoms. This complexation is augmented via the hydrogen bonds among water molecules and the negatively charged phosphate anions in conjunction with van der Waal interactions and the coordination of charge-balancing countercations. The orientation of ATP phosphate anions in the interprotein space occurs with van der Waal’s bonding of ATP’s adenine moiety interacting with the hydrophobic regions of protein surfaces; the hydrated ATP triphosphate extends via a ribose sugar into the interstitial water-laden interfacial space between adjacent proteins organizing the inter-protein aqueous medium (Figure 1). Thus, the hydrophobic regions of protein surfaces are cloaked with an organized lubricating ice-like matrix-on-protein surface. This matrix then maintains the separation of one protein from an adjacent protein, preventing the aggregation of proteins. This interprotein separation preventing protein aggregation then allows a protein to maintain its conformation and accomplish its designated function, whether it be enzymatic or structural. In contrast, if adjacent proteins are allowed to aggregate, they can become dysfunctional, as has been shown in a range of medical disorders [34], e.g., neurodegenerative diseases such as Alzheimer’s disease [35], as well as systemic disorders such as Type 2 diabetes mellitus [36]. These debilitating diseases are a major threat to human health.

Additional explanation of the presence of water molecules between adjacent ATP molecules is required. Crystallographic studies show that there are no water molecules within each ATP dimer complex [37], only between the triphosphate groups of adjacent ATP molecules. In contrast, the location of water molecules in the position depicted in our model (Figure 1) forms the basis for the layering of water molecules between ATP molecules. The polar triphosphate chain has been shown to strongly interact with water mediated through its multiple negative charges. Consider that given an adequate amount of ATP, the ATP moieties find each other and essentially adhere to the hydrophobic protein surfaces through the hydrophobic-hydrophobic interaction of the adenine moiety.

When ATP’s adenine moiety is bound to hydrophobic regions of protein surfaces and the hydrophilic triphosphate moiety is oriented into the interstitial water separating proteins, the interstitial water is bound to the ATP phosphates, and these phosphates interact with additional water molecules through hydrogen bonds. Theoretically, this establishes a second ice-like layer of water that is not bound directly to the ATP phosphates but to the water molecules that are bound directly to the ATP phosphates. This process continues outward from the protein-ATP surface forming an aqueous film with each successive water layer being less firmly bound to the ATP phosphate groups. As such, this water film becomes more fluid or water-like with further distancing of water molecules from the phosphate groups of the ATP molecule. In this more fluid-like delocalized region of the interstitial water there are increasingly randomly exchanging water molecules [38].

The crystalline lens contains a higher concentration of protein than any tissue in the body. The proteins are very close together; thus, the concept suggests that the presence of the ATP hydrotropic layer on adjacent proteins acts to stabilize the interstitial water layer as the proteins approach each other. This stabilization of adjacent proteins forms a liquid-crystalline interstitial gel-like layer, where the participating water molecules are more firmly bound into stable locations between the adjacent water layers. This is due to the polarizing effects of the negatively charged ATP phosphate groups (Figure 1). This gel-like layer, then, can act as a lubricating cushion between the adjacent protein molecules, keeping them separate. If there existed crystallographic data on the α-crystallins providing the distance between the protein surfaces, the number of water layers might be calculated.

### 3.6. Functions of ATP

Historically, the principal role or primary function traditionally ascribed to ATP is as the molecular reservoir for energy metabolism and the principal energy carrier and currency of living cells. This ascription appears to have occurred since the main source of ATP production in eukaryotes has been known to be mitochondrial. The mitochondrion is the organelle often cited as the ‘powerhouse’ of the cell. The phenomenon of a millimolar intracellular ATP concentration in eukaryotes appeared consistent, established in our review of various species [21]. This phenomenon permeates the entire phylogenetic tree [21]. There is a similarly high concentration of ATP in prokaryotic bacteria having no mitochondria. In fact, bacteria can exhibit high concentrations of ATP that are equivalent to those in eukaryotic tissues [21]. This observation presents an even greater enigma, since in bacteria, ATP is made in the cell membrane via proton pumps [39], and, being completely self-contained organisms, bacteria are known to exhibit high-energy demands. The concentration of ATP is not dependent on the metabolic activity of cells. In fact, in our study, cells with varying degrees of metabolic activity all appear to have similarly high millimolar concentrations of ATP [21].

Paradoxically, the concentration of ATP needed for metabolic function is known to require only a small fraction of the millimolar concentration of ATP reported. In fact, it appears that only a small portion of ATP is required for all the known functions of ATP (Table 1) combined. This is corroborated by Rice and Rosen [40] considering the Michaelis constant where only micromolar values are required for ATP-driven cellular processes. This raises the question as to why both micro-organisms (2.7 mM) [21] and intracellular lens tissue (2.3 mM) [26] have millimolar concentrations of ATP. Notably, the millimolar concentration of ATP is at a level three orders of magnitude greater than the micromolar concentration of ATP necessary for all metabolic intracellular functions in regular cellular metabolism. Our finding of a commonality of the millimolar intracellular concentrations of ATP across taxonomic domains is a foundational concept and is a fundamental biological feature of a living cell and ultimately its tissue and organ function [21].

## 4. Discussion

Although there has been increasing evidence to suggest a critical role for ATP in the maintenance of proteostasis in the cytoplasm [41], the paucity of studies linking ATP and protein aggregation and protein solubility is obvious from our review herein of the published literature indexed in PubMed. We suggest the following hypothesis: In the presence of high ATP concentrations, ATP functions as a biological hydrotrope resulting in the prevention of protein aggregation and the maintenance of protein solubility. Evidence for ATP functioning as a biological hydrotrope was first reported by Patel and coworkers [15] in cell and tissue homogenates, suggesting that at millimolar concentrations, ATP maintains protein solubility by exploiting the hydrotropic activity of ATP [40]. This hydrotropic function of ATP was further supported with our observation, using ^31^P NMR, of the relationship between intracellular ATP and water in an intact functioning living organ, the crystalline lens [19]. The hypothesis of ATP as a hydrotrope metabolite that binds with water is now supported by experiments using different technologies applied to cellular organelles [16], unicellular organisms [17,20], and Jurkat cells (an immortalized line of human T lymphocyte cells) [18].

Hayes and coworkers presented findings on the contribution of ATP to the maintenance of protein solubility using the nucleolar organelles from *Xenopus laevis* oocytes [16]. Employing reversed-phase-liquid-chromatography-electrospray ionization-tandem-mass-spectrometry analysis, Hayes et al. showed that the hydrotropic properties of ATP can affect naturally occurring protein aggregates [16].

Since ATP is required for the function of chaperones and proteases and ATP is required to fuel its multiple functions in facilitating protein folding and disaggregation, Pu and coworkers demonstrated that as ATP is exhausted and protein aggregation occurs, bacteria are unable to maintain regular metabolism and become dormant [17]. Pu et al. reported further that the ATP-facilitated protein aggregation proceeds in an ATP-dependent manner for bacterial regrowth. Experiments were conducted using mass spectrometry, and based on these findings, they proposed that ATP not only functioned as fuel for protein quality control but also as a hydrotrope to ensure proteostasis in a crowded cytoplasm [17].

As Takaine and coworkers point out [20], ATP levels have been shown to regulate the physicochemical properties of the cytoplasm, such as viscosity, macromolecular crowding, and liquid–liquid phase separation [15]. These studies on budding yeast strains and plasmids determined that high and stable ATP levels prevent aberrant intracellular protein aggregation in yeast (~4 mM ATP concentration) using the QUEEN-based single-cell ATP imaging technique [20]. This study demonstrated that cellular homeostasis ensures proteostasis and revealed that suppressing high ATP fluctuations of cellular ATP levels prevented cytotoxic protein aggregation [20]. A link between cellular energetics of ATP and protein-folding homeostasis is supported by the data presented [20].

Using multiplexed mass spectrometric data, Sridharan and coworkers interrogated ATP-mediated regulation of protein solubility on a proteome-wide scale [18]. The proteome profiling of Sridharan et al. revealed high-affinity interactions of ATP, where ATP is shown to have a wide-spread influence on protein complexes and their stability. These data provide quantitative insight into how ATP influences protein structure and solubility [18], which is consistent with the hydrotropic function of ATP.

ATP also may act in a synergistic manner. For example, Cyr observed that the combination of Saa2p, Ydj1p, and ATP almost completely suppressed rhodanese aggregation [42].

### 4.1. Strengths of the Hypothesis

The strength of this perspective is the presentation of data and hypotheses organized among multiple scientific disciplines ranging from elemental phosphorus chemistry to physics, biology, and medicine. Our understanding of the mechanisms of protein aggregation and disease, together with our knowledge of the enigmatically high concentration and function of the metabolite ATP [15,21], suggests that effective procedures for the prevention of disease are more than likely those that address the earliest events in preventing protein aggregation. Pharmaceutical intervention in the prevention of disease development might focus on maintaining high concentrations of ATP in the lens which represents an ideal model organ in which to study the reduction in the risk of protein aggregation. Such drug development might fortify the inherent defenses that maintain the naturally high concentrations of ATP to preserve protein solubility in an aqueous milieu for longer periods of time in a cell or tissue life cycle. Could these findings be applicable to cells of diverse phyla? The phylogenetic similarity in the concentration of ATP among cells, tissues, and organs is noteworthy [21].

### 4.2. Limitations of the Hypothesis

Limitations of our hypothesis may include the assumption that lens proteins remain in their nascent state throughout life, where ATP might indeed act as a hydrotropic factor. Additionally, our perspective is limited, since it does not address the implications of ATP as a molecular chaperone for ATP functions [43], or any of the other known functions of ATP (Table 1). Moreover, it is not our intention to pretend that lens proteins do not accrue many chemical modifications with time, for example, those associated with oxidation, deamidation, and racemization, that may be involved in or promote protein aggregation [44]. Intralenticular conditions might become favorable for phase separation, as well. An additional limitation is the paucity of literature regarding the interplay between ATP and protein aggregation in age-related vision-threatening diseases. Although there are literature references of protein alterations with aging, the specific interactions with ATP have not been addressed.

### 4.3. Future Directions

Studies designed to expand the understanding of the process of protein aggregation and its prevention increase our opportunity of interfering with the development of protein aggregates. Future directions for the delivery of molecular ATP or ATP-promoting pharmaceuticals to lenticular and retinal tissues should focus on the delivery systems of exogenous ATP or facilitation of intracellular production of ATP. Clinical efficacy using systemic drug delivery to the eye is attained with relatively high concentrations of a medicant when compared to localized topical or intracameral delivery. For example, using a corticosteroid for the treatment of uveitis, systemic delivery of efficacious concentrations of this pharmaceutical requires 60–100 mg. With regard to the delivery of pharmaceuticals, the anatomical site of the eye is privileged because of its accessible location in the body. As such, it permits both topical and intracameral delivery of pharmaceuticals. Other considerations must include molecular size, absorption/diffusion, and possible toxicity of ATP.

Topical delivery of a pharmaceutical via eyedrops can reach intraocular tissues including the lens and the retina. This is most evident considering the iatrogenic posterior subcapsular cataract secondary to topically delivered corticosteroids affecting lens cells and tissue [45]. Even in the case of the more posteriorly located retina, it has been established that topically applied corticosteroids and even non-steroidal anti-inflammatory pharmaceuticals can assist in the resolution of cystoid macular edema [46]. Although topical treatment delivered through the transcorneal route may be the safest, topically instilled applied ATP or pharmaceuticals promoting ATP production must be small enough to pass through the tear film and corneal tissue layers into the anterior chamber of the eye for accessibility to the lens and the retina.

Intracameral delivery of pharmaceuticals has been employed as an efficient route of delivery. Intracameral delivery includes intravitreal injection of fluid or implants or injection into the anterior chamber of implants. In contrast to topical drug delivery, where higher concentrations of ATP or its promoters are required, intravitreal injections can be employed. In this case, an injectable fluid or implant is delivered via the vitreous. Drug delivery is accomplished via syringe by entering the vitreous with a needle through the inferotemporal quadrant of the scleral wall of the eye, 4 mm posterior to the limbus. As such, a pharmaceutical containing ATP might be delivered to the vitreous humor in closer proximity to the retina.

As described above, since the eye is in a position of the body that is partially exposed, topical as well as intracameral delivery to attain effective tissue concentrations of a pharmaceutical is possible when attempting to deliver pharmaceuticals to the lens and retinal tissues. The eye offers an ideal opportunity to evaluate and study the effects of multiple drug delivery systems. The selection of appropriate ATP delivery to the lens or retina tissue [47,48], as noted above, may depend on the molecular charge, size, and associated molecular chemistry.

Regarding effective absorption/diffusion into the lens or retinal tissues, the molecular size and anionic charge of ATP must be considered. Since the uptake of the small-sized molecule must account for possible transport channels, the polyphosphate, magnesium, and calcium ions that line such channels are often associated with the ATP molecule. Also, there exists the possibility that ATP with its small size (approximates 1.4 nm; 500 Da) may simply be absorbed without the necessity for specific transport channels. Although delivery of the small ATP molecule into the lenticular and retinal tissues may promote maintenance of solubility of crystalline proteins and prevent formation of aggregates, the potential problems/difficulties in intracellular uptake cannot be ignored.

ATP delivery requires avoidance of potential toxicities and any adverse side effects of ATP on surrounding tissue structures when considering prescribing topical, intracameral, and systemic system delivery.

Considering the high millimolar concentration of ATP [21,26], the reports on the hydrotropic function of ATP [15,16,17,18,19,20] and our hypothesis support the likelihood that ATP is involved in the prevention of protein aggregation and maintaining protein solubility. Our hypothesis of ATP functioning as a hydrotrope relies on the delivery of ATP or promoters of ATP production to the proteins in the lens and retina target tissues. As such, some explanation is required concerning a better understanding of the lens organ and retinal macula in the context of protein aggregation in the three major vision-threatening diseases.

#### 4.3.1. Crystalline Lens and Age-Related Cataract

Worldwide, cataracts reportedly cause more than 40% of cases of blindness [49,50,51,52]. Although surgical lensectomy is an effective treatment for cataract disease, surgeries not only pose an economic burden but also potential complications such as postsurgical inflammation, intraocular lens dislocation, posterior capsular opacification [53], or even glare originating from the lenticular implant. The age-related cataract is an acquired opacification in the normally transparent crystalline lens caused by abnormal aggregation of lens crystallin proteins. Aggregation of misfolded lenticular proteins is the common etiology of age-related cataract [54,55], considered to be triggered by various post-translational modifications that have been described [56,57,58]. These misfolded proteins alter the interaction of lenticular proteins and significantly decrease the solubility and stability of crystallins, promoting the formation of aggregations and resultant lens opacification (cataractogenesis).

To briefly review, the crystalline lens functions as an organ refracting light to focus objects on the retina [59]. The refractive ability of the lens depends on its transparency which is due to the lack of intracellular organelles within the mature lens fiber cells and the highly ordered structure of the water-soluble crystallin proteins. The structural integrity and solubility of crystallins, enhanced by protein interactions, are important to maintain lens transparency, since these proteins last a lifetime without protein turnover [60]. These highly concentrated soluble α, β, and γ-crystallin proteins repel but closely approximate each other, and the hypothesis of ATP’s triphosphate moiety, and interaction with intracellular water presented in this perspective functions to separate adjacent crystallin proteins. This interprotein interaction of adjacent proteins and water allows for the glass-like or ice-like structure described above under the heading Hydrotropic Nature of ATP. By not aggregating, crystallin proteins play an important role in preventing the formation of lens aggregates. Lens aggregates can alter light transmission [61] and can ultimately appear as biomicroscopically visible opacification. As such, aggregation and insolubilization of crystallins cause the degeneration and opacification of the lens, inducing cataractogenesis [62,63,64]. Although cataract is a well-known disease, the mechanism(s) of eye lens protein aggregation is not well understood.

Considering our studies and studies by others supporting the function of ATP as a hydrotrope, ATP should be considered in future goals for the development of anti-cataract drugs. Not only is prevention of protein aggregation important, but novel pharmaceuticals must be also designed that can reverse lens opacification by dissolving the aggregation of crystallin proteins.

Recent studies have reported on several compounds that can prevent and dissolve protein aggregates in the lens which may lead to eventually offering a novel anti-cataract drug strategy [65,66]. Considering the hypothesis presented herein, ATP should be included among these compounds. Some substances have been shown to effectively reverse clouding lens transparency [65,67,68]. These findings are described elsewhere [69] and indicate that protein aggregation is not an endpoint and could be reversed with specific-small-molecule drugs. Development of such drugs may be the dawn of cataract pharmacopeia and cataract treatment [65]. These drugs must prevent aggregation of proteins [70], promote clearance of misfolded proteins, or both and may even work in concert with ATP. Issues regarding efficacy of these treatments must be considered as well as safety, which remain unknown. Further, research is needed to establish efficacy and safety profiles. However, ATP is a molecule already shown to be in high intracellular concentrations throughout the phylogenetic tree and in multiple tissues [21], and as such, its metabolic safety profile may already be considerable.

For effective drug delivery strategies, pharmacological future directions might include nanotechnology in combination with ATP that may offer even greater efficiency in drug delivery. This pharmacologic therapy may be an efficient method to reduce the cataract disease burden and its surgical costs [66]. Further work is required to explore exact mechanisms involved in cataractogenesis. Understanding the regulation of protein homeostasis in the lens will help to define new therapeutic targets and candidates for cataract treatments and thus impact the global morbidity associated with this major public health concern. Cataractogenesis and its accompanying decline in vision negatively impact quality of life. ATP functioning at high concentrations as a hydrotrope [19] might play a role in prevention.

#### 4.3.2. Presbyopia

Presbyopia is the most common eye disease. Presbyopia is a disease as it impairs normal visual function and is manifested by typically distinguishing signs and symptoms. It affects nearly everyone in the fifth decade of life experiencing a noticeable reduction in the rate and amplitude of lenticular accommodation. Uncorrected ability to focus on near objects is a worldwide major vision-related problem because of the absence or unavailability of adequate or affordable vision correction [71]. The onset of presbyopia is reported to occur earlier in women [72] and in individuals living in regions where they are subjected to increased ultraviolet radiation exposure [73]. The absence of adequate or uncorrected near vision results in a loss of human productivity [71,74] and ultimately has a negative impact on quality of life. The development of methods to prevent and postpone presbyopiogenesis and reverse presbyopia is needed. There are limitations of current corrective methods [75]. For example, bifocal or progressive multifocal spectacles may be associated with limitations of peripheral blurring, restriction of the visual field, and impaired depth perception. Taken together, such visual phenomena can increase the risk of missteps when ambulating with poor balance or loss of balance and falling. Monovision options where focus is fixed with contact lenses or intraocular lens implants restrict lenticular accommodative function. Contact lenses with required maintenance of lens care (with the exception of one-day use lenses), expense, reduced age-related manual dexterity limiting the handling of contact lenses, and age-related increasing dry eye symptoms may preclude their use. Surgical alternatives such as multifocal intraocular lens implants (IOL) can involve postsurgical complications and can be restrictive by prohibited costs and may not be without visual side effects.

The primary etiology of presbyopia with increased lens stiffness (hardness) is due to protein insolubilization and its consequent protein aggregation. In addition to age-related insolubilization and aggregation of lens proteins, there is loss of lens elasticity and an increase in the concentration of water-insoluble protein. With aging, there is an increase in lens organ stiffness [76], structurally reducing its ability to change shape and decreasing its accommodative function. Decreasing accommodative function results in the gradual loss in the ability of the lens to focus on near objects.

Although major lens protein modifications have been shown to include acylation [77], advanced glycation end-product formation [78], deamidation [79], sulfhydryl oxidation [80], racemization [81], and truncation [82], we recognize that in addition to these protein modifications, ATP has now been identified [15,19] as an allosteric modifier that can serve as a substrate. A series of experiments have been reported regarding promotion of protein solubility and reduction in stiffness in the lens [83,84,85,86], and considering there is a reduction in the concentration of ATP with aging, (1) the potential employment of ATP as a treatment offers an additional possibility.

#### 4.3.3. Age-Related Macular Degeneration

Age-related macular degeneration (AMD) is a major public health issue [87,88] and disease burden [89]. It is the most common cause of blindness in developed countries [90,91]. AMD is reportedly responsible for approximately 9% of all cases of blindness worldwide [92]. AMD is a complex, progressive neurodegenerative disease characterized by loss of central vision secondary to cellular dysfunction and cell loss in the macula. The macula located in the central retina is a specialized region of the retina responsible for achieving a high degree of visual acuity, and, therefore, the degenerative changes that result in a reduction in vision occur in AMD. This macular disease is characterized by pathological alteration in the retinal pigment epithelial (RPE) cells, Bruch’s membrane, and the choriocapillaris [93]. AMD is characterized by accumulation of protein aggregates of RPE intracellular lysosomal lipofuscin and extracellular drusen deposited between the basal lamina of the RPE and the inner collagenous layer of Bruch’s membrane. The histopathology of AMD [93] and mechanisms of protein aggregation in the RPE cells [11] has been reported. Due to constant chronic oxidative photostress, there is thought to be progressive degeneration and eventual death of the RPE cells [94,95]. The RPE cells being responsible for phagocytosis of photoreceptor outer segments result in dysfunction and loss of the overlying photoreceptors [92].

With aging, there is a concurrent increase in oxidative stress and consequently increased accumulation of lipid or protein aggregates (lipofuscin) in RPE cell lysosomes [96] and drusen formation in the extracellular space between the RPE and underlying infrajacent Bruch’s membrane. Each RPE cell in the macula is reportedly responsible for the phagocytosis of 30–40 photoreceptors [11], an enormous metabolic challenge for the lysosomal system of the RPE. With aging, there is a normal attrition of these nondividing RPE cells, further increasing the metabolic burden on neighboring cells [94,97]. The defects in the RPE in the macular area can cause such a reduction in visual acuity and contrast sensitivity that patients have difficulties in coping with the reduction in or the loss of useful vision leading to an inability to perform routine daily tasks.

The use of high concentrations of ATP in maintaining protein solubility, prevention of protein aggregation, and the potential of disaggregation of abnormal proteins in the retina may be important pharmacologically in age-related macular degenerative disease. The possibilities for employment of ATP in maintaining solubilization of proteins and prevention of aggregation in the treatment of macular disease should not be ignored.

## 5. Summary

Since aging is related to increased protein aggregation in both the lenticular and retinal tissues resulting in age-related cataract, presbyopia, and age-related macular degeneration, with increasing life expectancy, the number of people worldwide adversely affected by these diseases will increase. It should be understood that age-related protein aggregation and its potential prevention by ATP as described in our hypothesis may even assist in maintaining general health. The three major vision-threatening eye diseases all related to protein aggregation affect the function of the lenticular and retinal tissues, impairing the ability to perform even ordinary daily tasks.

In reviewing the literature regarding protein aggregation in the lens and the retina concerning all three of the above-described diseases, there is a notable paucity of investigations into the ATP metabolite and its relationship with the prevention of protein aggregation. This is especially surprising since ATP has such a high millimolar intracellular concentration that is not yet entirely accountable in the known functions of ATP (Table 1). Further experimentation regarding the importance of ATP on protein aggregation is required. The interaction of intracellular proteins with ATP’s hydrotropic function as a generic molecular class of molecules cannot be ignored.

With recognition that protein aggregation is related to the intracellular concentration of ATP, we hypothesize that (1) ATP serves a critical and foundational molecular function for organismal homeostasis and (2) the hydrotropic feature of ATP prevents pathological protein aggregation while maintaining protein solubility and cellular, tissue, and organismal function. As such, ATP plays a role as a hydrotrope in the prevention of protein aggregation and is involved in the most profound vision-threatening ophthalmic diseases leading to vision loss or blindness worldwide, i.e., age-related cataract, presbyopia, and age-related macular degeneration. This perspective organizes, and links, known information on ATP and protein aggregation with a fundamental unrecognized function of ATP in biology and medicine and its relationship to the three most vision-threatening ocular diseases affecting global eye health. This hydrotropic function of ATP cannot be ignored as a potential foundational change in our understanding of the importance of ATP in cellular, tissue, and organismal metabolism.

## Figures and Tables

**Table 1:** Functions of ATP.

A molecular carrier of intracellular energy;
The ultimate metabolic source of high-energy phosphate bonds;
The parent residue giving rise to vitamin dinucleotides and other cofactors;
An allosteric enzyme regulator for modulating protein activities;
The principal metabolite for cellular energy transduction mechanisms;
The transport of macromolecules, such as proteins, into and out of cells;
A phosphorylating agent in phosphate regulation of transmembrane proteins;
A source of the adenosine nucleotide, one of the 4 letters of the genetic code;
Hypothesized to be an intracellular hydrotropic functional molecule maintaining protein solubilization preventing protein aggregation.
